# Prescription of respiratory medication without an asthma diagnosis in children: a population based study

**DOI:** 10.1186/1472-6963-8-16

**Published:** 2008-01-22

**Authors:** Mira GP Zuidgeest, Liset van Dijk, Henriette A Smit, Johannes C van der Wouden, Bert Brunekreef, Hubert GM Leufkens, Madelon Bracke

**Affiliations:** 1 Department of Pharmacoepidemiology & Pharmacotherapy, Utrecht Institute for Pharmaceutical Sciences (UIPS), Utrecht University, The Netherlands; 2 Centre for Prevention and Health Services Research, National Institute for Public Health and the Environment (RIVM), Bilthoven, The Netherlands; 3 Netherlands Institute for Health Services Research (NIVEL), Utrecht, The Netherlands; 4 Department of General Practice, Erasmus MC – University Medical Center Rotterdam, The Netherlands; 5 Institute for Risk Assessment Sciences, Utrecht University, Utrecht, The Netherlands; 6 Julius Center for Health Sciences and Primary Care, University Medical Centre, Utrecht, The Netherlands

## Abstract

**Background:**

In pre-school children a diagnosis of asthma is not easily made and only a minority of wheezing children will develop persistent atopic asthma. According to the general consensus a diagnosis of asthma becomes more certain with increasing age. Therefore the congruence between asthma medication use and doctor-diagnosed asthma is expected to increase with age. The aim of this study is to evaluate the relationship between prescribing of asthma medication and doctor-diagnosed asthma in children age 0–17.

**Methods:**

We studied all 74,580 children below 18 years of age, belonging to 95 GP practices within the second Dutch national survey of general practice (DNSGP-2), in which GPs registered all physician-patient contacts during the year 2001. Status on prescribing of asthma medication (at least one prescription for beta2-agonists, inhaled corticosteroids, cromones or montelukast) and doctor-diagnosed asthma (coded according to the International Classification of Primary Care) was determined.

**Results:**

In total 7.5% of children received asthma medication and 4.1% had a diagnosis of asthma. Only 49% of all children receiving asthma medication was diagnosed as an asthmatic. Subgroup analyses on age, gender and therapy groups showed that the Positive Predictive Value (PPV) differs significantly between therapy groups only. The likelihood of having doctor-diagnosed asthma increased when a child received combination therapy of short acting beta2-agonists and inhaled corticosteroids (PPV = 0.64) and with the number of prescriptions (3 prescriptions or more, PPV = 0.66). Both prescribing of asthma medication and doctor-diagnosed asthma declined with age but the congruence between the two measures did not increase with age.

**Conclusion:**

In this study, less than half of all children receiving asthma medication had a registered diagnosis of asthma. Detailed subgroup analyses show that a diagnosis of asthma was present in at most 66%, even in groups of children treated intensively with asthma medication. Although age strongly influences the chance of being treated, remarkably, the congruence between prescribing of asthma medication and doctor-diagnosed asthma does not increase with age.

## Background

Asthma is a chronic inflammatory disease, associated with airway hyper responsiveness that leads to recurrent episodes of wheezing, shortness of breath and chest tightness [1]. Asthmatic complaints are relatively common in children [1-3]. The prevalence of asthma symptoms in children varies between global populations from less than 2% to approximately 33% of the population [4]. In the Netherlands in children from 2 to 15 years of age 4–12% experience shortness of breath and 5–20% experience chest wheezing. About 6,5% of 7–12 year olds has asthma [5]. Around one-third of all infants have one or more wheezing episodes in their first years of life [2].

There is substantial information, including guidelines, on how children with asthma symptoms should be treated, which kind of asthma medication should be used and which problems should be addressed [1,6,7]. However, studies describing actual use of asthma medication in children show ample variability in treatment patterns [3,8-12] and raise concern about over- and under treatment both in children with and without doctor-diagnosed asthma [8,10,11,13-16].

The interpretation of these findings differs with age of the child. When a pre-school child with asthmatic symptoms presents at the physician's office, a diagnosis of asthma is not easily made [17-21]. It is well known that wheezing at a young age may not only be due to asthma but also to other, more transient, respiratory conditions [18-23]. Different wheezing phenotypes have been described in children below the age of 6 of which transient early wheezing is the most common one counting for over 50% of wheezing children. This wheezing phenotype is often outgrown in the first 3 years of life and only a minority of wheezing children will develop persistent atopic asthma over time [1,24-26]. Despite this uncertainty in diagnosis, asthma medication is often prescribed to wheezing infants. Moreover, the response to asthma medication itself is widely used as a diagnostic tool to strengthen or reject the possible diagnosis of asthma [1,6,7,19,22].

According to current insights and diagnostics, the diagnosis of asthma can be assessed more accurately with increasing age. As can be deduced from both scientific literature and guidelines concerning treatment of children with asthmatic symptoms [1,6,7,27], the general consensus is that from the age of 5 to 6 years a diagnoses of asthma can be made with reasonable certainty. Therefore, theoretically, the congruence between use of asthma medication and doctor-diagnosed asthma is expected to increase with age.

However, previous studies have reported a mismatch between asthma medication use and a diagnosis of asthma [28-31]. Roberts et al. found that parents of 45.4% of children aged 0 to 17 receiving asthma medication, failed to report asthma [28]. And a study by Yeatts et al. showed a large group of undiagnosed frequent wheezers in children from 12 to 18 years of whom 12% used an inhaler in the past year [30]. In most of these studies the diagnosis of asthma was parental or self-reported, the study was not population based, a very specific age group was investigated or no extensive age group analysis has been performed.

We feel that a better comprehension of the congruence between use of asthma medication and doctor-diagnosed asthma is useful to improve asthma medication use in children. Therefore the aim of this study is to evaluate the relationship between prescribing of asthma medication and a diagnosis of asthma, as found in the GPs clinical record, in children and adolescents aged 0–17.

## Methods

### Setting and study population

This study has been conducted within the framework of the second Dutch national survey of general practice (DNSGP-2) which was carried out in 2001 by the Netherlands Institute for Health Services Research (NIVEL) and the National Institute for Public Health and the Environment (RIVM) [32,33]. The DNSGP-2 survey has been described in detail elsewhere [32,33]. In short, 195 general practitioners (GPs) in 104 practices, registered all physician-patient contacts during 12 consecutive months. The participating GPs formed a representative sample of the total population of Dutch GPs according to age and sex of the GP, region, and location of the practice (rural/urban; deprived area); only the percentage of single-handed GP practices was smaller in the DNSGP-2 study. The total practice population consisted of 391,294 patients at the start of the survey. The population characteristics corresponded very well to the Dutch population as a whole with respect to age, sex, and the type of health insurance.

The DNSGP-2 provides data on the nature and duration of GP-patient contacts, disease episodes, the diagnosis (coded using the International Classification of Primary Care [34]), the performed actions and all prescriptions made by the general practitioner. In the Netherlands all non-institutionalised inhabitants are registered in a general practice.

For the present study data from all 95 GP practices with adequate registration of physician-patient contacts and drug prescriptions was included. The study population consisted of all 74,580 children below the age of 18 years within these 95 practices.

### Measurements and analysis

#### Asthma medication

Prescription drugs were registered by the GP according to the Anatomical Therapeutic Chemical (ATC) Classification system [35]. The following of these were considered to be asthma medication: inhaled short acting beta2-agonists (SABA), oral short acting beta2-agonists, inhaled long acting beta2-agonists (LABA), inhaled corticosteroids (ICS), inhaled cromones, and montelukast. All children with at least one prescription for one of these medicine groups in the year 2001 were included in the study.

Asthma medication users were further subdivided into four asthma therapy groups, which were most common in our study population: (i) monotherapy with short acting beta2-agonists (inhaled or oral), (ii) monotherapy with inhaled corticosteroids, (iii) combination therapy of these two medication groups (9.7% of the children in this group also received one or more other asthma medicines) and (iv) all other therapies.

To be able to differentiate between one-time asthma medication users (where the medication itself might be used as a diagnostic tool to strengthen or reject the diagnosis of asthma) and the more chronic users we also made a subdivision into children with (i) only one prescription, (ii) two prescriptions and (iii) three or more prescriptions during the study period. All medications prescribed on the same day were considered to belong to one prescription.

#### Doctor-diagnosed asthma

During the study period GPs registered all contacts with their patients, including face-to-face consultations as well as telephone consultations and repeat prescriptions. Every single health problem presented within a consultation was coded by the GP using the International Classification for Primary Care (ICPC) [34]. When a patient presented two complaints within one consultation, these were coded as two separate (sub)consultations. Therefore it is known for every patient how often he or she contacted the GP and for which health problems. GPs were trained during an intensive course on coding practices and problems.

In this study all children who, in the year under study, contacted the GP with a health problem subsequently coded as ICPC R96 (asthma) were considered to have doctor-diagnosed asthma.

#### Data analysis

To quantify the congruence between prescription of asthma medication and doctor-diagnosed asthma we determined Sensitivity and Positive Predictive Value (PPV) of asthma medication use as a predictor for doctor-diagnosed asthma. Based on the general consensus that from the age of 5 to 6 years a diagnosis of asthma can be made with reasonable certainty we divided the study population into two age groups: children below the age of 6 and children aged 6 years and older and analysed whether the found congruence changes. We repeated these analyses in subgroups of age, gender, asthma therapy and number of prescriptions. We also performed these analyses increasing the cut-off point for defining 'asthma medication use' from 1 to a minimum of 2 prescriptions, this way excluding possible trial medication. The results from these analyses are added as online repository material (see additional file 1).

Statistical analyses were performed using SAS, version 9.1 (SAS Institute, Cary, NC).

## Results

General characteristics of the study population are presented in Table 1. The study population was similar to the Dutch population with respect to gender and age groups. Prescription of asthma medication within our study population was 7.5% in the year 2001. In the same episode a diagnosis of asthma was present in the GPs' clinical record for 4.1% of the study population. Thus prescribing of asthma medication was twice as common as doctor-diagnosed asthma.

**Table 1:** Patient characteristics of the study population

	**Total study population (74,580)**		**Asthma medication users (5,605)**		**Children with doctor-diagnosed asthma (3,064)**	
	**N**	**%**	**N**	**%**	**N**	**%**

Gender						
Male	38,267	51.3	3,160	56.4	1,775	57.9
Female	36,313	48.7	2,445	43.6	1,289	42.1

Age (years)						
0–2	9,030	12.1	1053	18.8	540	17.6
3–5	12,690	17.0	1300	23.2	689	22.5
6–8	13,357	17.9	1004	17.9	589	19.2
9–11	13,521	18.1	853	15.2	490	16.0
12–14	13,087	17.6	738	13.2	445	14.5
15–17	12,895	17.3	657	11.7	311	10.2

Other respiratory problems						
shortness of breath	367	0.5	240	4.3	101	3.3
wheezing	176	0.2	144	2.6	46	1.5
acute URTI*	6307	8.5	1122	20.0	530	17.3
acute bronchitis/bronchiolitis	2269	3.0	1002	17.9	406	13.3
pneumonia	679	0.9	254	4.5	129	4.2
allergic rhinitis	1983	2.7	394	7.0	223	7.3

Number of contacts with GP						
0	22784	30.5	240	4.3	0	0
1–2	26725	35.8	1314	23.4	658	21.5
≥3	25071	33.6	4051	72.3	2406	78.5

Oral corticosteroid use	337	0.5	188	3.4	143	4.7

At least 1 parent with doctor-diagnosed asthma^	3106	4.3	483	8.9	312	10.5

* URTI = Upper Respiratory Tract Infection

^ Based on data from 96% of the study population due to missing values (N = 71712)

An overview of the applied therapies, stratified by age groups is presented in Table 2. We see that prescription of asthma medication changed with age. The prescribing of asthma medication was highest in children age 0–2 years (11.7%) and steadily declined with rising age, to 5.1% in the group of 15–17 year old children. Although differences could be observed in the applied therapies, there were no obvious trends with age. Monotherapy with inhaled short acting beta2-agonist and the combination of this drug with inhaled corticosteroids were the most applied therapies in all age groups. Of all children receiving asthma medication 52.9% received medication on only one occasion.

**Table 2:** Prescription of asthma medication by age group and type of medication

	**Age groups**						
	0–2	3–5	6–8	9–11	12–14	15–17	Total

	n = 9,030	n = 12,690	n = 13,357	n = 13,521	n = 13,087	n = 12,895	n = 74,580

Prescription of asthma medication	11.7	10.2	7.5	6.3	5.6	5.1	7.5

Therapy groups, %							
SABA	36.9	24.9	28.0	32.2	40.4	40.5	32.7
ICS	19.5	22.9	23.1	20.5	16.3	19.6	20.7
SABA + ICS	35.0	45.3	43.4	40.0	36.7	30.9	39.4
Other medicines	8.6	7.0	5.5	7.3	6.6	9.0	7.2

1 prescription	58.1	52.5	48.3	52.3	52.6	53.7	52.9
2 prescriptions	20.2	21.1	21.9	21.0	19.7	20.2	20.8
≥3 prescriptions	21.7	26.5	29.8	26.7	27.8	26.0	26.3

SABA = short acting beta2-agonists; ICS = inhaled corticosteroids

**Figure 1:**
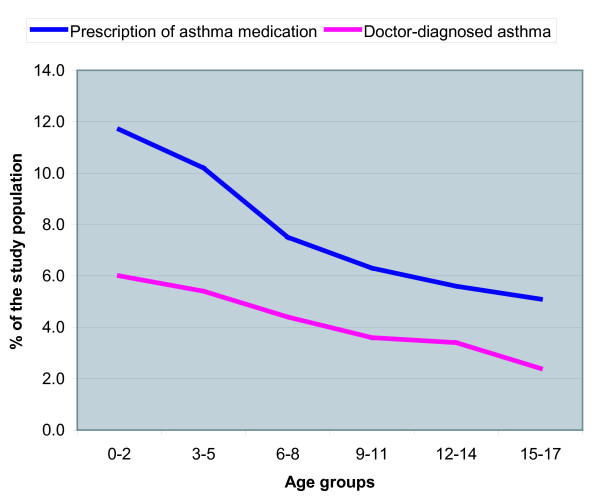
The course of prescription of asthma medication and doctor-diagnosed asthma with age.

In Figure 1 we plotted the course of prescribing of asthma medication and doctor-diagnosed asthma with age. We found that both prescribing of asthma medication and doctor-diagnosed asthma declined with age. Initially prescribing of asthma medication seems to decline more rapidly with age than a diagnosis of asthma. Taking into account only the first 3 age groups one would expect the lines of prescribing and diagnosis to eventually cross each other. However, after age 6–8 the lines run virtually parallel, thus the gap between prescribing and diagnosing does not resolve. Moreover, approximately twice as many children received asthma medication than a diagnosis of asthma both in age group 0–2 and in age group 15–17.

**Figure 2:**
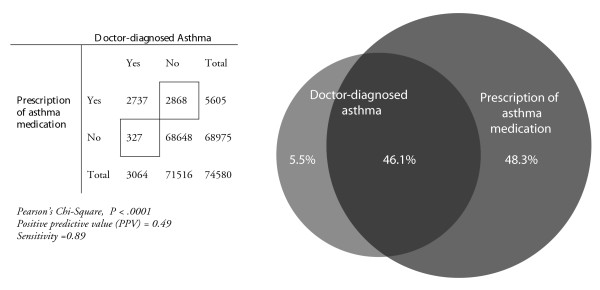
Congruence between asthma medication use and doctor-diagnosed asthma within the total study population.

The congruence between prescribing of asthma medication and doctor-diagnosed asthma for the overall study population is shown in two ways in Figure 2. The sensitivity shows that when selecting all asthma medication users, 89% of children with doctor-diagnosed asthma would be included in this selection. In other words, almost all children with doctor-diagnosed asthma were prescribed asthma medication. On the other hand, indicated by a PPV of 0.49, when selecting a child with asthma medication, this child would have doctor-diagnosed asthma in only 49% of the cases. Children receiving asthma medication without having doctor-diagnosed asthma had a registered diagnosis for other respiratory diseases (including acute upper respiratory infections, acute bronchitis, bronchiolitis and allergic rhinitis) or only a complaints diagnosis (including dyspnoea, wheezing and cough) during the registration period in respectively 45.4% and 21.9% of the cases.

Based on the general consensus that from the age of 5 to 6 years a diagnosis of asthma can be made with reasonable clinical certainty, we divided the study population into two age groups: children below the age of 6 and children aged 6 years and older. In Figure 2 we showed that there is an obvious age difference with respect to the number of children treated with asthma medication and diagnosed as being asthmatics. However, the congruence between these two measures does not differ between younger (<6) and older children (≥6). Below 6 years of age 46% of the treated children had a diagnosis of asthma and in the older age group this was 51%.

Further subgroup analyses are shown in Table 3. Boys were more often treated with asthma medication and diagnosed with asthma than girls, but the PPV and sensitivity were similar. Between medication groups the PPV differed significantly: children on combination therapy were more likely to have doctor-diagnosed asthma. Also children who received asthma medication on more than one occasion had a greater chance of being diagnosed as asthmatics, with a PPV rising from 0.38 for children with one prescription to 0.66 for children with at least three prescriptions. The results from the analyses after changing our cut-off point for defining 'asthma medication use' from 1 to a minimum of 2 prescriptions are added as online repository material (see additional file 1).

**Table 3:** Subgroup analysis of the congruence between prescription of asthma medication and doctor-diagnosed asthma.

	**N**	**Asthma medication use, %**	**Doctor-diagnosed asthma, %**	PPV*	**Sensitivity**
Total population	74,580	7.5	4.1	0.49	0.89

Male	38,267	8.3	4.6	0.50	0.89
Female	36,312	6.7	3.5	0.47	0.90

<6 yrs	21,720	10.8	5.7	0.46	0.88
≥6 yrs	52,860	6.2	3.5	0.51	0.90

SABA only		2.5	4.1	0.38	^
ICS only		1.6	4.1	0.42	
SABA + ICS		3.0	4.1	0.64	

1 prescription		4.0	4.1	0.38	^
2 prescriptions		1.6	4.1	0.54	
≥3 prescriptions		2.0	4.1	0.66	

* All Pearson's Chi-Square p-values < .0001

^ The sensitivities for subgroups of asthma medication users are not shown, since they are highly dependent on the percentual contribution of the subgroups to the total group of asthma medication users and are therefore not very informative and, by definition, low.

Looking at gender differences we found that the difference between boys and girls lies mainly in the number of children treated with combination therapy and diagnosed with asthma (both higher in boys) but not in congruence between medication and diagnosis. Only the PPVs of ICS use only and of one prescription only were slightly higher in boys (0.45 and 0.40 respectively) than in girls (0.38 and 0.35 respectively).

Within the two age classes (<6 years and ≥6 years) separately, again no major differences in congruence were found between subgroups of gender, asthma therapy and number of prescriptions. The results for the older age group separately are added as online repository material (see additional file 2). The sensitivity remained high throughout the entire analyses, children with an asthma diagnosis received asthma medication in 87–91% of the cases. In children below the age of 6 gender differences were more pronounced. Girls this age were treated less than boys (4.5% versus 6.7%) and when treated, they were less likely to get diagnosed with asthma (PPV of 0.42 versus 0.49). In the older age group these gender differences were not present, 3.2% of girls was treated and 3.8% of boys and the PPVs were 0.50 and 0.51 respectively. This also shows that in boys the PPV was the same in both age groups (0.51) whereas this differed for girls (PPV 0.42 for girls <6 and 0.50 for girls ≥6). The lowest overall PPV found was for girls below 6 years of age. Within this group 58% of children with asthma medication did not have doctor-diagnosed asthma.

## Discussion

The congruence between asthma medication use and doctor-diagnosed asthma can be considered from two angles. One way is to determine whether all children with a diagnosis of asthma receive treatment. In our study an overall high sensitivity of 0.89 shows that indeed a high percentage of children diagnosed with asthma receive asthma medication. The other way, investigating whether children using asthma medication have an asthma diagnosis, we find that overall only 49% of all children receiving asthma medication has doctor-diagnosed asthma (i.e., 2,868 children receive asthma medication without a diagnosis of asthma). This discrepancy could be due to many factors, including over treatment, off-label use, under diagnosis and use of asthma medication as a diagnostic tool.

We hypothesised that the congruence between prescription of asthma medication and doctor-diagnosed asthma would increase with age. We expected to find more undiagnosed children and children with one prescription only in children using asthma medication below the age of 6, because of diagnostic difficulties at this age and the use of medication as a diagnostic tool. Most children who wheeze at this age suffer from transient or self-limiting disease and might receive medication to lessen complaints but do not receive the label 'asthma'. From age 6 the diagnosis of asthma can be made with more certainty and the children receiving asthma medication are expected to be the 'real asthmatics' and thus have a matching diagnosis of asthma. We find that indeed both prescribing of asthma medication and doctor-diagnosed asthma are influenced by age. However, they both decline with rising age and the high sensitivity and low positive predictive value (PPV) found in the overall study population was consistent throughout the different age groups.

In subgroup analyses on gender and age we found only minor differences in PPV. The largest difference is between young girls (<6 years) and older girls (≥6) with 42% versus 50% receiving treatment without a diagnosis of asthma.

The PPV differs significantly between subgroups of therapy. Children on combination therapy have a much higher chance of having doctor-diagnosed asthma (PPV 0.64) than do children using short-acting beta2-agonists only (PPV 0.38). Also the number of prescriptions for asthma medication strongly influences the relationship with doctor-diagnosed asthma. However, the PPV never comes close to 1, thus no matter which subgroup you select, there is always a substantial number of children present that is treated with asthma medication without being diagnosed as an asthmatic.

The number of children using asthma medication and the declining trend with age found in our study is consistent with results from other studies [11,36] as is the number of children with doctor-diagnosed asthma[37].

Recent guidelines state that the first 2 treatment steps to manage asthma for children age 6 and older are the following: start with inhaled short acting beta2-agonists (SABA) as needed and add a low-dose inhaled corticosteroids (ICS) when symptoms are more frequent and/or worsen periodically. And although the evidence is not as strong in children age 5 years and younger, which precludes detailed treatment recommendations, these same 2 initial steps are recommended for this age group [1,6,7]. For the major part the therapies applied in our study population seem to be in line with these guidelines. However, there is quite a large group of children receiving ICS without receiving a prescription for SABA in that same year. This could indicate that there is some overtreatment with ICS. Another explanation could be that children had very little need for reliever medication and still used SABA dispensed in the previous year. Indeed, although the guidelines state that reliever medication should always be provided for quick relief of symptoms, reducing or eliminating the need for reliever treatment is both an important goal and a measure of success of treatment [1].

Our finding that 89% of children diagnosed with asthma receive asthma medication differs from other studies, in which many children with a diagnosis of asthma did not receive medication (ranging from 21.8 to 64.4%) [12,28,30,31]. However, these studies used parental-reported doctor-diagnosed asthma from questionnaire data, which have been shown to differ substantially from a diagnosis of asthma derived from the GPs clinical records in the sense that self-reported asthma renders many more 'asthmatics' than do the GP's records [38]. Despite this higher number of 'asthmatics' these studies do show similar results with respect to the percentage of children being treated without an asthma diagnosis. This percentage is lower than our finding (20.9%) in only one study, but comparable, ranging from 40.0% to 47.3%, in the other four studies [3,12,28,31,39]. One previous study used medical and pharmacy claim data instead of questionnaire data and found that children receiving asthma medication without a diagnosis of asthma have considerable morbidity and health care utilisation. The authors conclude that better recognition of paediatric asthma is warranted [29]. Although this study was not population based (but made use of an administrative claims based dataset) and mainly focuses on the economic impact of undiagnosed children dispensed asthma medication, without detailed subgroup analyses, their finding that 40% of children used asthma medication without evidence of a documented doctor-diagnosed claim for asthma is in line with our own findings.

The current study has some limitations. First, we do not have information on specialist care. In the hypothetical case that a GP continues asthma medication initiated by a specialist without registering the diagnosis this would add to the group of children being treated without a diagnosis of asthma. However, very few children in our study population were referred to a child lung physician (0.4% of children using asthma medication) or even to a paediatrician in general (5.4% of children using asthma medication). Second, in this study asthma diagnoses as registered by the GP are taken into account, and although often used as a gold standard in studies, this might not be the true reflection of asthmatics in the study population. Third, some overestimation of medication use might be present since not all prescribed medication is filled in the pharmacy. Since our interest lies in the relationship between the two GP based actions of diagnosing and prescribing (Does the GP base prescribing of medication on his decision whether or not to diagnose a child with asthma?), these last two limitations are of only relative importance to our study.

## Conclusion

Several conclusions can be drawn from the work presented here. Firstly, less than half of all children prescribed asthma medication had a registered diagnosis of asthma. Secondly, detailed subgroup analyses show that a diagnosis of asthma is present in at most 66% of children; even in children treated extensively with asthma medication, such as children prescribed inhaled corticosteroids or receiving asthma medication on at least three occasions within one year. Lastly, although age strongly influences the chance of being treated, remarkably, the congruence between prescribing of asthma medication and doctor-diagnosed asthma does not improve with age. Further research is needed to determine what causes this discrepancy and whether this is grounds for changing asthma medication use in children.

## Competing interests

As an employee of Erasmus MC – University Medical Center Rotterdam, Johannes C van der Wouden has received research funding from GlaxoSmithKline and ARTU Biologicals.

All other authors declare that they have no competing interests.

## Authors' contributions

MGPZ participated in designing the study, performed the analyses and drafted the final manuscript. MB participated in the design of the study and helped in the interpretation of the data. LvD helped in the preparation and interpretation of the data.

HGML participated in the design of the study and helped in the interpretation of the data. All authors critically reviewed draft versions of the manuscript. All authors read and approved the final manuscript.

## Pre-publication history

The pre-publication history for this paper can be accessed here:



## Supplementary Material

Additional file 1 Table 4. Subgroup analysis of the congruence between prescription of asthma medication and doctor-diagnosed asthma, with a cut-off for asthma medication use of at least 2 prescriptions Click here for file

Additional file 2 Table 5. Subgroup analysis of the congruence between prescription of asthma medication and doctor-diagnosed asthma for children age 6 and older. Click here for file
