# Supplementary material for: Prescription of respiratory medication without an asthma diagnosis in children: a population based study
Source: BMC Health Serv Res. 2008 Jan 22;8:16. doi: 10.1186/1472-6963-8-16 (PMC2245932; doi:10.1186/1472-6963-8-16)
Supplement: Additional file 2 — Table 5. Subgroup analysis of the congruence between prescription of asthma medication and doctor-diagnosed asthma for children age 6 and older. [file 1472-6963-8-16-S2.pdf]

Table 5 - Subgroup analysis of the congruence between prescription of asthma medication and doctor-diagnosed asthma for children age 6 and older.

	N	Asthma medication use, %	Doctor-diagnosed asthma, %	PPV*	Sensitivity
Total population	52,860	6.2	3.5	0.51	0.90
Male	26,959	6.6	3.8	0.51	0.89
Female	25,902	5.7	3.2	0.50	0.91
SABA only		2.1	3.5	0.41	^
ICS only		1.2	3.5	0.40	
SABA + ICS		2.4	3.5	0.66	
1 prescription		3.2	3.5	0.41	^
2 prescriptions		1.3	3.5	0.55	
≥ 3 prescriptions		1.7	3.5	0.66	

* All Pearson's Chi-Square p-values $< .0001$

^ The sensitivities for subgroups of asthma medication users are not shown, since they are highly dependent on the percentual contribution of the subgroups to the total group of asthma medication users and are therefore not very informative and, by definition, low.
